# AC and AG dinucleotide repeats in the *PAX6* P1 promoter are associated with high myopia

**Published:** 2009-11-05

**Authors:** Tsz Kin Ng, Ching Yan Lam, Dennis Shun Chiu Lam, Sylvia Wai Yee Chiang, Pancy Oi Sin Tam, Dan Yi Wang, Bao Jian Fan, Gary Hin-Fai Yam, Dorothy Shu Ping Fan, Chi Pui Pang

**Affiliations:** 1 Department of Ophthalmology & Visual Sciences, The Chinese University of Hong Kong, Hong Kong S.A.R.; 2 Present affiliation: Department of Ophthalmology, Harvard Medical School, Massachusetts Eye and Ear Infirmary, Boston, MA

## Abstract

**Purpose:**

The *PAX6* gene, located at the reported myopia locus *MYP7* on chromosome 11p13, was postulated to be associated with myopia development. This study investigated the association of *PAX6* with high myopia in 379 high myopia patients and 349 controls.

**Methods:**

High myopia patients had refractive errors of –6.00 diopters or greater and axial length longer than 26 mm. Control subjects had refractive errors less than –1.00 diopter and axial length shorter than 24 mm. The P1 promoter, all coding sequences, and adjacent splice-site regions of the *PAX6* gene were screened in all study subjects by polymerase chain reaction and direct sequencing. *PAX6* P1 promoter-luciferase constructs with variable AC and AG repeat lengths were prepared and transfected into human ARPE-19 cells prior to assaying for their transcriptional activities.

**Results:**

No sequence alterations in the coding or splicing regions showed an association with high myopia. Two dinucleotide repeats, (AC)_m_ and (AG)_n_, in the P1 promoter region were found to be highly polymorphic and significantly associated with high myopia. Higher repeat numbers were observed in high myopia patients for both (AC)_m_ (empirical *p* = 0.013) and (AG)_n_ (empirical *p* = 0.012) dinucleotide polymorphisms, with a 1.327-fold increased risk associated with the (AG)_n_ repeat (empirical *p* = 0.016; 95% confidence interval: 1.059–1.663). Luciferase-reporter analysis showed elevated transcription activity with increasing individual (AC)_m_ and (AG)_n_ and combined (AC)_m_(AG)_n_ repeat lengths.

**Conclusions:**

Our results revealed an association between high myopia and AC and AG dinucleotide repeat lengths in the *PAX6* P1 promoter, indicating the involvement of *PAX6* in the pathogenesis of high myopia.

## Introduction

Myopia, one of the most common refractive errors of the eye worldwide, is an important public health issue, especially in Asia, because of its higher prevalence in Asians than in other populations [1]. The progression of myopia in Chinese children in Hong Kong and Singapore is also much higher than in Caucasians [2,3]. In Hong Kong, the prevalence of myopia in Chinese schoolchildren aged 11–16 was 36.7%, according to a 2004 report, which is several times higher than among Caucasian children of similar ages [4]. The prevalence of high myopia, defined as a refractive error equal to or greater than –6.00 diopters (D), is also higher in Chinese than in Caucasians [5,6]. Individuals with high myopia are more prone to develop serious ocular complications, such as retinal detachment, glaucoma, premature cataracts, and macular degeneration, which may lead to visual impairment or even blindness [7-10].

Myopia is a complex disorder. Multiple interacting environmental and genetic causes are implicated. Myopia development in schoolchildren has been attributed to environmental factors, such as near work, reading habits, and school achievement [3,11,12]. In addition, high heritability of refractive errors has been observed in dizygotic and monozygotic twin studies [13-17]. Family and sibling studies have shown that children of myopic parents have greater chances of developing myopia than those with nonmyopic parents [11,18]. Twenty-four chromosomal loci have been identified for myopia: Xq28 (*MYP1)* [19], 18p11.31 (*MYP2*) [20,21], 12q21-31 (*MYP3)* [22], 7q36 (*MYP4)* [23], 17q21-22 (*MYP5)* [24], 22q37.1 (*MYP6)* [25], 11p13 (*MYP7*) [26], 3q26 (*MYP8)* [26], 4q12 (*MYP9)* [26], 8p23 (*MYP10)* [26], 4q22-q27 (*MYP11)* [27], 2q37.1 (*MYP12)* [28], Xq23 (*MYP13)* [29], 1p36 (*MYP14)* [30], 10q21.2 *(MYP15)* [31], 5p15.33-p15.2 (*MYP16*) [32], 7p15 (*MYP17*) [33,34], 14q22.1-q24.2 (*MYP18*) [35], 15q12-13 [36], 21q22.3 [37], 12q24 [38], 4q21 [38], 9q34.11 [39] and 2q37 [40]. Among them, * MYP1–5*, *11–13*, *16*, and *18* are linked to high myopia, and *MYP2*, *11*, *13*, *16*, and *18* are found in the Chinese population. Some candidate genes have been postulated for myopia, such as *TGIF* [41], *HGF* [42], *MMP3* [43], *MMP9* [43], *COL1A1* [44], *COL2A1* [45], *TGFB1* [46], *TGFB2* [47], *LUM* [48], and *CMET* [49].

A genome-wide scan in dizygotic twins revealed a susceptibility locus for myopia on chromosome 11p13 [26]. The *PAX6* gene at this locus, a member of the paired-domain PAX family, has been postulated as a candidate gene for myopia. *PAX6* is expressed in the human eye [50] and plays an evolutionarily conserved role in ocular development [51-53]. *PAX6* mutations are associated with ocular disorders, such as aniridia (OMIM 106210), cataracts (OMIM 604219), Peters anomaly (OMIM 604229), and optic nerve hypoplasia (OMIM 16550). *PAX6* encodes a transcriptional regulator containing the DNA-binding paired domain, paired-type homeodomain, and COOH-terminal transactivation domain. The Pax6 protein regulates cell adhesion molecules, cell-to-cell signaling molecules, hormones, and structural proteins [54] through interactions with transcription factors such as Mitf [55] and Sox2 [56]. Transcription of *PAX6* is regulated by at least two promoters, P0 and P1 [57-60]. Within the P1 promoter (promoter B in Okladnova et al. [59]), two dinucleotide repeats, (AC)_m_ and (AG)_n_, are located about 1 kb from the transcription start site [58] and are highly polymorphic in Caucasians. The poly AC and poly AG repeats are independently polymorphic [60]. Luciferase analysis in Cos-7 cells has shown that the longer the combined length of the AC and AG repeats, the higher the transcriptional activity, implying that the length of this dinucleotide repeat might influence the transcriptional activity of promoter B, or P1, and subsequently the transcription of *PAX6*.

Pax6 levels are tightly controlled. Both overexpression and haploinsufficiency lead to abnormal phenotypes [61-63]. Polymorphisms or mutations in the *PAX6* promoter could influence *PAX6* expressions that ultimately lead to a disease phenotype. However, although *PAX6* has been postulated to be a candidate gene for myopia, several studies in Caucasian populations could not find an association between *PAX6* and myopia [26,45,64]. Still, an Australian study suggested *PAX6* mutations might be associated with high myopia [65]. Intronic sequence alterations (SNPs) in *PAX6* have been reported to associate with high myopia in Han Chinese nuclear families [66] and with extreme myopia in a Taiwan Chinese population [67], but not in Caucasians. To attest the association between *PAX6* and high myopia, we should look for mutations that may affect *PAX6* expressions. We therefore screened for sequence alterations in the P1 promoter, coding exons, and adjacent splice-site regions of *PAX6* in unrelated high myopia patients and control subjects. We also examined transcriptional effects of dinucleotide repeats within the P1 promoter in cultured human APRE-19 cells by a luciferase-reporter assay and predicted the presence of transcription factor binding sites within the repeats.

## Methods

### Study subjects

We recruited 379 unrelated Han Chinese patients with high myopia at the Hong Kong Eye Hospital. They were given complete ophthalmoscopic examinations. None of them had known diseases predisposing them to myopia, such as Stickler or Marfan syndromes. Their refractive errors were equal to or greater than –6.00 D, and their axial length was longer than 26 mm. We also recruited 349 unrelated Chinese control subjects who visited the hospital for ophthalmic examinations. They had no eye diseases except senile cataracts and slight floaters. All of them had refractive errors of less than –1.00 D and axial length shorter than 24 mm. The study protocol was approved by the Ethics Committee for Human Research at the Chinese University of Hong Kong and was in accordance with the tenets of the Declaration of Helsinki. Informed consent was obtained from the study subjects after explanation of the nature and possible consequences of the study.

### *PAX6* genotyping

The whole blood specimens (5 ml) from all the patients and controls were collected in EDTA tube and stored at -80 °C for fewer than two months. Genomic DNA was extracted (QIAamp DNA kit; Qiagen, Hiden, Germeny) according to the supplier’s instructions. All samples were screened for sequence alterations in the P1 promoter region flanking –3,433 to –118, coding exons, and intron-exon boundaries of *PAX6* (ENSG00000007372 and ENST00000241001; Ensembl genome browser) by polymerase chain reaction (PCR) with primer sets [61]. PCR was performed in a final volume of 25 μl containing 1X PCR buffer (Invitrogen™ Life Technology, Carlsbad, CA), 1.5 mM MgCl_2_, 0.2 mM of dNTP (Roche, Indianapolis, IN), 0.2 mM of each primers, 0.5 U of Platinum® Taq DNA polymerase (Invitrogen). After the initial denaturation at 95 °C for 2 min, 40 PCR cycles were conducted: 95 °C for 45 s, 57 °C for 45 s and 72 °C for 45 s. The final extension lasted for 5 min at 72 °C. Direct sequencing was performed using a BigDye Terminator Cycle Sequencing Reaction Kit (v3.1, Applied Biosystems, Foster City, CA) on an ABI 3130XL capillary DNA sequencer (Applied Biosystems).

### Construction of *PAX6* P1 promoter-luciferase constructs

A 1,851 bp genomic fragment (from –1278 to +573) containing the *PAX6* P1 promoter was cloned into an empty pGL3-Basic vector, pGL3 (Promega, Madison, WI) between the SacI and BglII sites (OriGene Technologies, Rockville, MD). Constructs with different repeat lengths were generated. Genomic DNA from the study subjects was amplified by PCR (forward primer 5'-ACA CAC AGA TGA CCG GTG G-3'; reverse primer 5'-AAG CCT AGG CCG AGA GGA-3'). AgeI and AvrII digested products were ligated into a linearized pGL3-Basic vector containing the P1 promoter (pGL3-Pax6p). A positive control construct was made by cloning a pCMV5 promoter [68] into the pGL3-Basic vector (pGL3-pCMV). All constructs were verified by direct sequencing.

### Cell culture and transfection

The human retinal pigment epithelial cell line ARPE-19 (American Type Culture Collection, Manassas, VA) [69] was cultured in Dulbecco’s modified Eagle’s medium and F-12 nutrient mixture supplemented with 10% fetal bovine serum (Gibco BRL, Rockville, MD). Cells were plated in 60 mm tissue culture dishes at a density of 2–3×10^5^ cells/dish one day before transfection. At 60–80% confluence, cells were transfected with 2 μg luciferase constructs in 6 μl FuGene HD (Roche) transfection reagent per dish. Empty pGL3 and pGL3-pCMV were used as negative and positive controls, respectively. At 36 h after transfection, cell lysates were extracted using Cell Culture Lysis Reagent (Promega, Madison, WI) for immunoblotting.

### Immunoblotting

The denatured cell lysates of the transfected cells were resolved on 10% SDS-polyacrylamide gel and electro-transferred to nitrocellulose membranes for probing with a rabbit polyclonal primary antibody against firefly luciferase (Sigma-Aldrich, St. Louis, MO) and a secondary antibody against rabbit IgG conjugated with horseradish peroxidase (Jackson Immuno Res., West Grove, PA). The chemiluminescence was detected by an enhanced chemiluminescence system (Amersham Pharmacia, Cleveland, OH) and quantified by ChemiDoc (BioRad, Hercules, CA). Normalized luciferase intensities were calculated by dividing the quantified luciferase intensities by the housekeeping β-actin intensities. Triplicates were performed.

### Statistical analysis

The χ^2^ test or Fisher exact test was used to compare the allele and genotype frequencies of SNPs in patients and control subjects. For the comparison of (AC)_m_ and (AG)_n_ repeat alleles and genotypes between high myopia patients and control subjects, the χ^2^ test was performed using the CLUMP program (version 2.3) [70]. For multiple testing corrections, 10,000 Monte Carlo permutations were chosen to simulate the empirical significance levels of the statistics produced by the program, resulting in an empirical p-value. Due to low frequencies of some alleles, and in order to determine whether the transcriptional activities were affected by the thresholds, (AC)_m_ and (AG)_n_ repeats were collapsed into groups for association study and immunoblotting analysis [71,72]. The risk of high myopia was also determined by odds ratio using the χ^2^ test. Activity of each allelic construct was expressed relative to (AC)_20_(AG)_6_. One-way ANOVA and independent T-testing were used to compare the means among (AC)_m_ groups and between (AG)_n_ repeats, respectively. SNP-trait association, odds ratio calculation, and immunoblotting analysis were performed on SPSS version 16.0 (SPSS Science, Chicago, IL). Significance was defined as p < 0.05.

### Transcription factor binding site prediction

The DNA sequence of the cloned *PAX6* P1 promoter was used to predict transcription factor binding sites. The Transcription Element Search System (TESS: University of Pennsylvania, Philadelphia, PA) [73,74] was used to predict the transcription factors that would bind to the region of the dinucleotide repeats in the *PAX6* P1 promoter. Predictions for different lengths of dinucleotide repeats were also performed. As in the statistical analysis for immunoblotting, (AC)_20_(AG)_6_ was set as a reference.

## Results

In our study cohort, high myopia patients had a mean age of 39.52±14.96 years and a male-to-female ratio of 1.2:1. Refractive errors ranged from –6.00 to –30.00 D. For the controls, the mean age was 64.85±14.85 years, with a male-to-female ratio of 1.6:1. There was no significant difference in the sex ratio between high myopia patients and controls.

Two sequence changes were identified in coding exons with the intron-exon boundary of *PAX6*. One novel heterozygous silent variant, 678A>G (R67R), was found in one high myopia patient, and a noncoding sequence change, rs667773, was found in both patients and controls. Allelic and genotypic frequencies of both polymorphisms showed no significant difference (p > 0.05) between patients and controls (data not shown). Within the P1 promoter region, 20 polymorphisms were identified, with no significant difference in frequencies between patients and controls: –186C>T, –215G>A, –242G>A, –263A>G, –292A>G, –331A>G, –337A>T, –354A>G, –382G>A, –407G>A, –409G>A, –692A>G, –758C>T, –782A>G, –933C>G, –3050C>A, –3070C>A, –3078A>G, –3090C>T, and –3282T>C (data not shown). For -186C>T, -292A>G, -331A>G, -933C>G, and -3282T>C, each SNP was only found in 1 high myopia patient. Therefore, they were statistically not significant under Pearson’s χ2 test (p > 0.05).

Within the *PAX6* P1 promoter, two dinucleotide repeats, (AC)_m_ and (AG)_n_, were observed about 1 kb from the transcription start site, both highly polymorphic (Table 1). The AC repeats ranged from 16 to 26 in high myopia patients and from 7 to 26 in control subjects, while 5 to 8 AG repeats were observed in patients and 4 to 8 in controls. The median numbers of AC and AG repeats were 20 and 6, respectively, in both patients and controls. Distribution of the allele frequencies was slightly skewed in patients for both AC and AG repeats. Allele frequencies of the AC and AG repeats were significantly different between patients and controls (empirical p = 0.013 and 0.012, respectively; Table 1). Because the frequencies of some of the alleles were low, the AC and AG repeats were collapsed into groups. The grouped repeat lengths were longer in patients than in controls (empirical p = 0.016 for (AC)_m_ and empirical *p* = 0.016 for (AG)_n_; Table 2). In terms of risk analysis, individuals with (AG)_7-8_ repeats had a 1.327-fold increased risk of developing high myopia compared with the those with (AG)_4-6_ repeats (empirical p = 0.016; 95% confidence interval = 1.059–1.663). Both grouped AC and grouped AG genotypes were significantly different between high myopia patients and control (empirical p = 0.004 and 0.039, respectively; Table 3).

**Table 1:** Allelic frequencies of *PAX6* P1 promoter dinucleotide repeats in high myopia (HM) and control subjects.

**(AC)_m_ repeat**	**Allelic count (%)**		**Empirical p-value**	**(AG)_n_ repeat**	**Allelic count (%)**		**Empirical p-value**
	**HM n=750**	**Control n=678**			**HM n=758**	**Control n=698**	
(AC)_7_	0 (0.0)	1 (0.1)	0.013	(AG)_4_	0 (0.0)	1 (0.1)	0.012
(AC)_15_	0 (0.0)	2 (0.3)		(AG)_5_	45 (5.9)	51 (7.3)	
(AC)_16_	10 (1.3)	9 (1.3)		(AG)_6_	464 (61.2)	458 (65.6)	
(AC)_17_	43 (5.7)	41 (6.0)		(AG)_7_	218 (28.8)	176 (25.2)	
(AC)_18_	80 (10.7)	67 (9.9)		(AG)_8_	31 (4.1)	12 (1.7)	
(AC)_19_	100 (13.3)	138 (20.4)					
(AC)_20_	155 (20.7)	134 (19.8)					
(AC)_21_	149 (19.9)	99 (14.6)					
(AC)_22_	161 (21.5)	138 (20.4)					
(AC_)23_	29 (3.9)	33 (4.9)					
(AC)_24_	13 (1.7)	13 (1.9)					
(AC)_25_	6 (0.8)	2 (0.3)					
(AC)_26_	4 (0.5)	1 (0.1)					

**Table 2:** Allelic frequencies of *PAX6* P1 promoter grouped dinucleotide repeats, (AC)_m_ and (AG)n, in high myopia (HM) and control subjects.

**Grouped (AC)_m_ repeat**	**Allelic count (%)**		**Empirical p-value**	**Grouped (AG)_n_ repeat**	**Allelic count (%)**		**Empirical p-value**
	**HM n=750**	**Control n=678**			**HM n=758**	**Control n=698**	
(AC)_Below 20-22_	233 (31.1)	258 (38.1)	0.016	(AG)_4-6_	509 (67.2)	510 (73.1)	0.016
(AC)_20-22_	465 (62.0)	371 (54.7)		(AG)_7-8_	249 (32.8)	188 (26.9)	
(AC)_Above 20-22_	52 (6.9)	49 (7.2)					

**Table 3:** Genotypic frequencies of *PAX6* P1 promoter grouped dinucleotide repeats in high myopia (HM) and control subjects.

**Grouped (AC)_m_ genotype**	**Genotypic count (%)**		**Empirical p-value**	**Grouped (AG)_n_ genotype**	**Genotypic count (%)**		**Empirical p-value**
	**HM n=375**	**Control n=339**			**HM n=379**	**Control n=349**	
(AC)_Below 20-22_ / (AC)_Below 20-22_	16 (4.3%)	40 (11.8%)	0.004	(AG)_4-6_ / (AG)_4-6_	173 (45.6%)	192 (55.0%)	0.039
(AC)_Below 20-22_ / (AC)_20-22_	178 (47.5%)	149 (44.0%)		(AG_)4-6_ / (AG)_7-8_	163 (43.0%)	126 (36.1%)	
(AC)_Below 20-22_ / (AC)_Above 20-22_	24 (6.4%)	29 (8.6%)		(AG)_7-8_ / (AG)_7-8_	43 (11.3%)	31 (8.9%)	
(AC)_20-22_ / (AC)_20-22_	130 (34.7%)	103 (34.7%)					
(AC)_20-22_ / (AC)_Above 20-22_	26 (6.9%)	16 (4.7%)					
(AC)_Above 20-22_ / (AC)_Above 20-22_	1 (0.3%)	2 (0.6%)					

**Figure 1:**
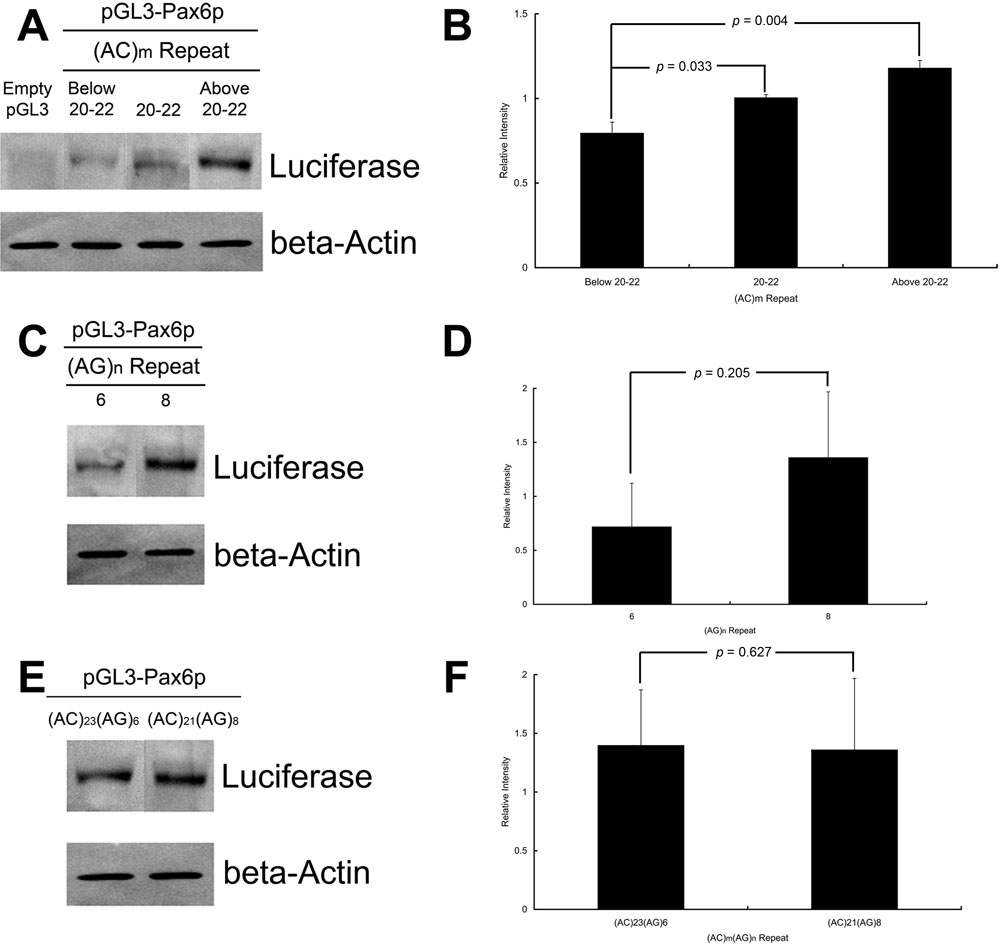
Transcriptional activity of dinucleotide repeats in the *PAX6* P1 promoter. A 1,851 bp genomic fragment (from –1278 to +573) containing the *PAX6* P1 promoter with different dinucleotide repeats was cloned into an empty pGL3-Basic vector (pGL3) and transfected into ARPE-19 cells. The activity of each allelic construct is expressed relative to the construct (AC)_20_(AG)_6_. Data are represented as mean±SD for five independent experiments. **A** and **B:** Immunoblotting results and a bar chart show relative luciferase activity for grouped (AC)_m_ repeats with a stable (AG)_6_. **C** and **D**: Immunoblotting results and a bar chart show relative luciferase activity for (AG)_n_ repeats with (AC)_21_. **E** and **F**: Immunoblotting results and a bar chart show relative luciferase activity for combined (AC)_m_(AG)_n_ repeats.

We found that the dinucleotide repeats affected the transcriptional activity of the *PAX6* P1 promoter (Figure 1). For a given (AG)_n_ repeat length, elevated transcriptional activity was observed with increasing length of (AC)_m_ repeats (p = 0.004, one-way ANOVA; post-hoc tests adjusted by Tukey HSD: (AC)_Below20–22_ versus (AC)_20–22_, p = 0.033; and (AC)_Below20–22_ versus (AC)_Above20–22_, p = 0.004; Figure 1A,B). Similarly, at a given (AC)_m_ repeat length, transcriptional activity of (AG)_8_ was increased when compared with (AG)_6_, although the increase was not significant, likely due to the substantial standard deviation (p = 0.205, independent T-test; Figure 1C,D). For combined repeats of the same length, transcriptional activity of (AC)_23_(AG)_6_ was similar to that of (AC)_21_(AG)_8_ (p = 0.627, independent T-test; Figure 1E,F). Thus, both AC and AG repeats contributed to the transcriptional activity of the *PAX6* P1 promoter.

**Figure 2:**
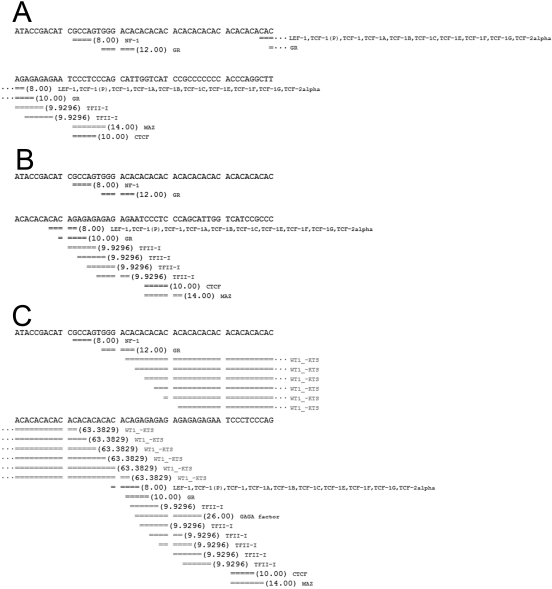
Transcription factor binding site prediction for dinucleotide repeats in the *PAX6* P1 promoter. The cloned *PAX6* P1 promoter DNA sequence was used to predict transcription factor binding sites. Predicted transcription factor binding sites around the region of the dinucleotide repeats are shown, and different lengths of AC and AG repeats are assessed. As in the immunoblotting analysis, [(AC)_20_(AG)_6_] was set as a reference. **A**: Predicted transcription factor binding sites for (AC)_15_(AG)_4_ are shown. **B**: Predicted transcription factor binding sites for (AC)_20_(AG)_6_ are shown. **C**: Predicted transcription factor binding sites for (AC)_26_(AG)_8_ are shown.

Our luciferase-reporter analysis showed that transcription activity increased with AC and AG repeat length. This phenomenon may be due to influences of transcription factor binding sites within this region. Thus, we used (AC)_20_(AG)_6_ as a reference and predicted one binding site for T-cell factor/Lymphoid enhancer factor family transcription factors, one glucocorticoid receptor binding site, and four transcription factor (TF) II-I binding sites (Figure 2B). With decreasing AG repeat lengths, the T-cell factor/Lymphoid enhancer factor and glucocorticoid receptor sites were unchanged, but the TFII-I sites were reduced. Only two predicted TFII-I sites were observed in (AC)_15_(AG)_4_ (Figure 2A). No alteration was observed with a decrease in AC repeat lengths. Accordingly, more TFII-I sites were predicted with increasing AG repeat lengths. Multiple sites for Wilms’ tumor transcription factor without lysine-threonine-serine [Wt1(–KTS)] were observed with an increase in AC repeat length, and one GAGA factor binding site appeared with an increase in AG repeat length. In (AC)_26_(AG)_8_, six TFII-I sites, six Wt1(–KTS) sites, and one GAGA factor site were predicted (Figure 2C).

## Discussion

We found no myopia mutations in the coding regions and splice sites in *PAX6* in our cohort of Chinese high myopia patients*.* Some SNPs were detected in the P1 promoter, exon 7, and intron 10, but these were not statistically significant (data not shown). In a recent report, two intronic SNPs (rs3026390 and rs3026393, located in introns 12 and 13, respectively) have been shown to be associated with high myopia in Han Chinese nuclear families [66]. SNP rs667773, located in intron 10, is in the same linkage disequilibrium block with rs3026390 and rs3026393 [66]. However, in our study, no significant association was found for rs667773 between high myopia patients and controls, which was consistent with a previous case-control association study in a Taiwan Chinese population [67]. The discrepancy might be due to the much lower minor allele frequency of rs667773 (0.137) than of rs3026390 and rs3026393 (0.472 and 0.493, respectively) [66]. Other studies have suggested that rs667773, as a neural polymorphism, is an unlikely cause of overt phenotypes such as aniridia [75,76].

The (AC)_m_(AG)_n_ dinucleotide repeat sequence, located about 1 kb from the transcription start site of the *PAX6* P1 promoter, is highly polymorphic. The AC dinucleotide polymorphism ranged from 18 to 31 repeats and AG ranged from 5 to 7 repeats in a Caucasian population [60]. In our Chinese cohort, the AC repeats ranged from 7 to 26 and the AG repeats from 4 to 8 (Table 1). The allele size of the AG repeats was similar in Caucasians and Chinese, but the AC repeat length was longer in Caucasians. Notably, one (AC)_7_ allele was found in a control subject, far from the common range of repeats between 15 and 26. In addition, many of the dinucleotide repeats were heterozygous in both poly AC and poly AG repeats (AC: 55.3% in controls and 75.9% in patients; AG: 42.9% in controls and 53.4% in patients). The observed heterozygosity rate was 65% in a Caucasian population [60]. Although the allele number in that study was defined as combined units of AC and AG repeats instead of independent AC and AG alleles, the trend of heterozygosity was similar to that in our work. These two dinucleotide repeats are, therefore, highly polymorphic both in Caucasians and in Chinese.

The *PAX6* P1, containing CCAAT boxes and a TATA-like box, is likely a real promoter [58-60]. We evaluated the influence of (AC)_m_(AG)_n_ dinucleotide repeats on *PAX6* P1 promoter activity by a luciferase-reporter assay and examined the effects of repeat lengths as obtained from our high myopia patients and controls. Since retinal pigment epithelium (RPE) has been shown to have *PAX6* P1 promoter activity [57], we used an RPE cell line, ARPE-19, for transfection. Immunoblotting showed that longer lengths of (AC)_m_ have a significant trend of increasing luciferase expression compared with shorter lengths (Figure 1A,B), although this was not observed for (AG)_n_, likely due to the substantial standard deviation (Figure 1C,D).

We confirmed that transcriptional activity of (AC)_23_(AG)_6_ was similar to that of (AC)_21_(AG)_8_ (Figure 1E,F), suggesting that both (AC)_m_ and (AG)_n_ dinucleotide repeats within the *PAX6* P1 promoter contribute to transcriptional activity and might work cooperatively as an unit. Previous studies on luciferase-reporter assays assessed the promoter activity invisibly by a luminometer [60,77]. In our study, we monitored the luciferase-reporter assay by immunoblotting using a commercially available antibody against firefly luciferase and luciferase overexpression by pGL3-pCMV as a positive control. There are technical advantages to this method. The promoter activity could be visualized, and co-transfection with another normalizing vector was not required, as the luciferase intensity could be directly normalized with the housekeeping protein, assuming the same transfection efficiency among the constructs. The limitation of the luciferase-reporter assay is that the effect of the dinucleotide repeats on the transcriptional activity was performed using RPE cells from normal controls, which might not truly reflect the situation in high myopia unless the experiment were performed using cells from a highly myopic individual.

Since levels of Pax6 are tightly controlled, small and seemingly insignificant changes in the levels of Pax6 may lead to significant phenotypic consequences [78]. Moreover, the Pax6 protein could upregulate *PAX6* P1 promoter activity [77]. Results of our genotyping and promoter activity analyses indicate that longer lengths of dinucleotide repeats increase the expression of *PAX6*, which increases the risk of high myopia. This postulation may be supported by several assertions: (1) *PAX6* gene expression has been shown to be significantly higher in the retinas of optical defocused eyes than in contralateral eyes in the rhesus monkey [79], and expression of *PAX6* was also increased in posthatch chicken eyes with form-deprivation myopia [78]. (2) In another study, the number of dividing retinal progenitor cells, of which *PAX6* is a marker, was highly correlated with axial elongation of the eye, resulting in myopic refractive errors in primates with form-deprivation myopia [80]. (3) Pax6 has been shown to transactivate insulin promoters [81] and promote proinsulin processing [82]. As insulin is a strong stimulator of axial myopia in chicks [83], elevated *PAX6* expression may increase the risk of developing myopia through increased expression of insulin. Chronic hyperinsulinemia has been proposed as a key player in the pathogenesis of juvenile-onset myopia [84]. Although Pax6 also transactivates the glucagon promoter [81], which is a “stop” for myopia [85], insulin might overcome the effects of glucagon in the development of myopia [86].

The transcription factor binding site prediction (Figure 2) showed that an increase in AC repeat length created additional Wt1(–KTS) binding sites, while an increase in AG repeat length created TFII-I and GAGA factor binding sites. If the AG repeat length was reduced, TFII-I sites were also reduced. Wt1(–KTS) is necessary for normal retina formation in mice [87], while TFII-I is a signal-induced multifunctional transcription factor that plays a key role in the regulation of cell proliferation [88]. Moreover, the GAGA factor, a transcription activator, is activated by epidermal growth factors, platelet-derived growth factors, and insulin [89]. These growth factors could regulate *PAX6* transcription through the GAGA factor binding site.

In summary, we found no association between polymorphisms in the *PAX6* coding region and high myopia in our Hong Kong Chinese cohort. Two dinucleotide repeats, AC and AG, in the *PAX6* P1 promoter were associated with high myopia. These two repeats were also associated with the elevation of *PAX6* P1 promoter activity, and hence an increase in the transcriptional activity of *PAX6.* Our results provide evidence for the role of *PAX6* in the pathogenesis of high myopia.
